# Investigating the performance and cost-effectiveness of the simple ultrasound-based rules compared to the risk of malignancy index in the diagnosis of ovarian cancer (SUBSONiC-study): protocol of a prospective multicenter cohort study in the Netherlands

**DOI:** 10.1186/s12885-015-1319-5

**Published:** 2015-06-26

**Authors:** Evelyne MJ Meys, Iris JG Rutten, Roy FPM Kruitwagen, Brigitte F Slangen, Martin GM Bergmans, Helen JMM Mertens, Ernst Nolting, Dieuwke Boskamp, Regina GH Beets-Tan, Toon van Gorp

**Affiliations:** 1 Department of Obstetrics and Gynecology, Maastricht University Medical Centre (MUMC+), P. Debyelaan 25, 6202 AZ Maastricht, The Netherlands; 2 GROW - School for Oncology and Developmental Biology, Maastricht University Medical Centre (MUMC+), P. Debyelaan 25, 6202 AZ Maastricht, The Netherlands; 3 Department of Obstetrics and Gynecology, Laurentius Ziekenhuis Roermond, Mgr. Driessenstraat 6, PO Box 920,, 6040 AX Roermond, The Netherlands; 4 Department of Obstetrics and Gynecology, Orbis Medical Centre Sittard, Dr. H. van der Hoffplein 1, PO Box 5500,, 6130 MB Sittard, The Netherlands; 5 Department of Obstetrics and Gynecology, St. Jans Gasthuis Weert, Vogelsbleek 5, PO Box 29,, 6000 AA Weert, The Netherlands; 6 Department of Obstetrics and Gynecology, VieCuri Venlo, Tegelseweg 210, PO Box 1926,, 5900 BX Venlo, The Netherlands; 7 Department of Radiology, Maastricht University Medical Centre (MUMC+), P. Debyelaan 25, 6202 AZ Maastricht, The Netherlands

**Keywords:** Ovarian cancer, Ultrasound, Risk of malignancy index, Simple ultrasound-based rules, Subjective assessment, Diffusion weighted imaging, MRI, Diagnosis

## Abstract

**Background:**

Estimating the risk of malignancy is essential in the management of adnexal masses. An accurate differential diagnosis between benign and malignant masses will reduce morbidity and costs due to unnecessary operations, and will improve referral to a gynecologic oncologist for specialized cancer care, which improves outcome and overall survival. The Risk of Malignancy Index is currently the most commonly used method in clinical practice, but has a relatively low diagnostic accuracy (sensitivity 75–80 % and specificity 85–90 %). Recent reports show that other methods, such as simple ultrasound-based rules, subjective assessment and (Diffusion Weighted) Magnetic Resonance Imaging might be superior to the RMI in the pre-operative differentiation of adnexal masses.

**Methods/Design:**

A prospective multicenter cohort study will be performed in the south of The Netherlands. A total of 270 women diagnosed with at least one pelvic mass that is suspected to be of ovarian origin who will undergo surgery, will be enrolled. We will apply the Risk of Malignancy Index with a cut-off value of 200 and a two-step triage test consisting of simple ultrasound-based rules supplemented -if necessary- with either subjective assessment by an expert sonographer or Magnetic Resonance Imaging with diffusion weighted sequences, to characterize the adnexal masses. The histological diagnosis will be the reference standard. Diagnostic performances will be expressed as sensitivity, specificity, positive and negative predictive values and likelihood ratios.

**Discussion:**

We hypothesize that this two-step triage test, including the simple ultrasound-based rules, will have better diagnostic accuracy than the Risk of Malignancy Index and therefore will improve the management of women with adnexal masses. Furthermore, we expect this two-step test to be more cost-effective. If the hypothesis is confirmed, the results of this study could have major effects on current guidelines and implementation of the triage test in daily clinical practice could be a possibility.

**Trial registration:**

ClinicalTrials.gov: registration number NCT02218502

## Background

Ovarian cancer is the second most common gynecologic malignancy [1]. In 2008 it was the seventh leading cause of cancer deaths in women worldwide [1, 2]. Estimating the risk of malignancy is essential in the management of adnexal masses. Patients with a malignancy should undergo an appropriate staging procedure or debulking surgery carried out in specialized surgical centers. This is associated with a better median survival [3]. Vice versa, patients with a benign lesion may be managed conservatively or with minimal invasive surgery in non-specialized hospitals. This will limit morbidity and avoid unnecessary costs: laparoscopic surgery is associated with less blood loss, shorter hospital stay, and fewer postoperative complications with an improved quality of life and faster return to preoperative functioning [4].

**Fig. 1:**
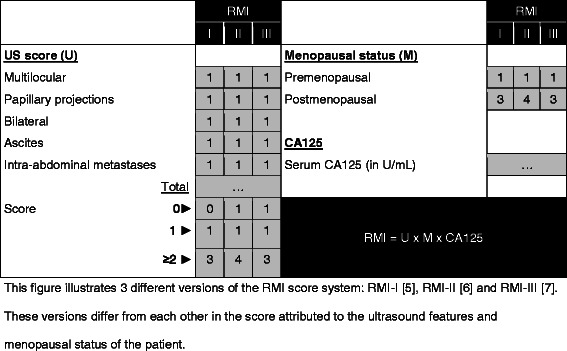
Schematic presentation of three different RMI score algorithms. This figure illustrates 3 different versions of the RMI score system: RMI-I [5], RMI-II [6] and RMI-III [7]. These versions differ from each other in the score attributed to the ultrasound features and menopausal status of the patient

There are several methods to distinguish benign from malignant adnexal masses. The commonly most used method in clinical practice is the Risk of Malignancy Index (RMI) [5–7]. The RMI is an easy to use scoring system that is recommended by many national guidelines in the differential diagnosis of ovarian masses. The RMI combines ultrasound variables, menopausal status and serum CA125 into a score used to predict the risk of ovarian cancer before surgery (Fig. 1). The reported sensitivity and specificity of RMI at a cut-off value of 200 are 75–80 % and 85–90 %, respectively [8]. This results in an incorrect diagnosis (false positive or false negative) in one out of five women with an adnexal mass. These patients therefore receive inappropriate treatment, potentially leading to increased morbidity and/or mortality.

The ‘simple ultrasound-based rules’ (simple rules) is another method to differentiate between benignity and malignancy. This method uses different morphological ultrasound features of adnexal masses (without including menopausal status or serum CA125 measurement). It includes 10 rules (Table 1); five rules to predict malignancy (M-rules) and five rules to predict a benign tumor (B-rules). If both or none of the M- and B-rules are met the test is inconclusive [9,10]. Simple rules are applicable in approximately 80 % of patients with an ovarian mass and in these cases a sensitivity of 95 % and a specificity of 91 % is achieved in previous studies [11].

**Table 1:** Benign and malignant ultrasonic features used in simple ultrasound-based rules as proposed by Timmerman et al. [9]

10 Simple ultrasound-based rules	
B-features (for predicting a benign tumor)	
B1	Unilocular
B2	Presence of solid components, of which largest solid component has largest diameter <7 mm
B3	Presence of acoustic shadows
B4	Smooth multilocular tumor with largest diameter <100 mm
B5	No blood flow (color score 1)
M-features (for predicting a malignant tumor)	
M1	Irregular solid tumor
M2	Presence of ascites
M3	At least four papillary structures
M4	Irregular multilocular solid tumor with largest diameter ≥100 mm
M5	Very strong blood flow (color score 4)

*Rule 1*: If ≥ 1 M-features are present in absence of B-feature(s), the mass is classified as malignant

*Rule 2*: If ≥ 1 B-features are present in absence of M-feature(s), the mass is classified as benign

*Rule 3*: If both M-features and B-features are present, or if no B- or M-features are present, the result is inconclusive and a second stage test is recommended

In adnexal masses for which the simple rules yield an inconclusive result (unclassifiable masses), subjective assessment by Gray-scale and color Doppler ultrasound imaging by an experienced ultrasound examiner can be used as a second stage test to achieve an optimal diagnostic performance. This subjective assessment is also called ‘pattern recognition’ [12]. The downside of subjective assessment is that it is experience dependent. Nevertheless, subjective assessment by an expert sonographer seems to be superior to any scoring system or mathematical model when classifying adnexal masses [10, 13, 14]. However, it is neither feasible nor efficient that every patient would have to undergo an expert ultrasonography. Therefore, this method is very well suited as a second stage test. In cases where the simple rules were inconclusive (i.e., masses that are difficult to diagnose) subjective assessment was used successfully with a sensitivity of 91 % and a specificity of 93 % [10]. Several individual reports have confirmed that this two-step triage test is superior to the RMI, especially in terms of sensitivity [15–17].

Magnetic Resonance Imaging (MRI) with diffusion weighted sequences is another option for second stage testing of unclassifiable masses. The use of MRI – when interpret by specialized radiologists - also seems to be superior to RMI in the preoperative identification of adnexal masses. A meta-analysis performed by Dodge et al. resulted in an overall sensitivity of 92 % and a specificity of 88 % [18]. The test holds as an advantage that an MRI can not only distinguish a benign from a malignant mass, but can also detect possible metastases in case of a malignancy. Furthermore, MRI can help to select patients who might be more appropriately managed by neoadjuvant chemotherapy [19].

### Objectives

The primary objective of the SUBSONiC-study (Simple Ultrasound Based ruleS to differentiate OvariaN Cysts) is to test the hypothesis that the simple rules, supplemented -in case of an inconclusive result- with either subjective assessment by an expert sonographer or MRI, will give better diagnostic accuracy than the RMI and therefore will improve the management of women with adnexal masses. Since the RMI is now widely used, any result in favor of the triage test has the potential to alter future clinical practice and reduce costs. Based on the results a cost-effectiveness analysis will be performed.

Secondary objectives are to perform subgroup-analyses for premenopausal and postmenopausal women and to compare the diagnostic accuracy of subjective assessment by an experienced ultrasound examiner with MRI for those cases where the simple ultrasound-based rules were inconclusive. Furthermore, the study aims to assess various forms of interobserver-agreement: in the interpretation of MRI images between radiologists; in the interpretation of simple ultrasound-based rules between the primary ultrasound and the expert ultrasound; and in the subjective assessment between the primary ultrasound and the expert ultrasound. Last objective is to perform translational research and validate new biomarkers in the diagnosis of ovarian cancer.

## Methods/Design

### Study design

We will perform a prospective multicenter cohort study in the south of The Netherlands; four regional hospitals will participate in the study (Laurentius hospital Roermond, Orbis medical center Sittard, St. Jans hospital Weert and VieCuri Venlo) together with a tertiary referral center (Maastricht University Medical Centre +). In total, 270 patients will be included within a timeframe of two years. The study will end once the last patient’s final diagnosis based on histology is known.

### Study population

Female patients 18 years of age or older are eligible to participate in the study if they are diagnosed in one of the participating centers with at least one pelvic mass that is suspected to be of ovarian origin, and are to undergo surgery in order to obtain a final histological diagnosis. Exclusion criteria are as follows: pregnancy, age under 18 years, a prior bilateral oophorectomy, insufficient or missing data, no informed consent, surgery did not take place or takes place more than 120 days after RMI and simple ultrasound-based rules are performed, and/or the patient is not able or willing to travel to the center hospital for additional diagnostic procedures.

### Study parameters

The primary research question is the comparison of diagnostic test accuracy between the currently used RMI and a new two-step triage system for the correct differentiation between malignant and benign adnexal masses. Main study parameters are sensitivity, specificity, positive predictive value, negative predictive value and positive and negative likelihood ratios. Sensitivity is defined as the percentage of women with ovarian cancer diagnosed with a malignancy by the RMI and the two-step test, respectively. The positive predictive value is defined as the percentage of patients with a positive test result having malignant disease. The final diagnosis will be based on histology (gold standard).

Based on the results, a cost-effectiveness analysis will be performed.

### Study procedures

Both RMI and simple rules will be performed in the regional hospitals and center hospital by general gynecologists during the same ultrasound scan. For collection of these variables a routine transvaginal ultrasound is sufficient. Transabdominal ultrasonography will be added if the mass is too large to be seen entirely by using only transvaginal ultrasonography. Gray scale and color Doppler ultrasound live images will be used to obtain all morphologic and blood flow variables to characterize each mass by RMI and by simple rules. The ultrasound will be performed by a general gynecologist or a trainee supervised by a general gynecologist. The sonographer will not be blinded for the serum CA125 level since this is needed to calculate the RMI.

Only when the simple rules are inconclusive the patient will be referred to the center hospital for the second stage tests; i.e., subjective assessment and MRI. From previous publications it can be deducted that this will be in approximately 20 % of patients.

Subjective assessment will be performed by an expert ultrasound examiner. The ultrasound examiner will be a level III sonographer according to the guidelines of the European Federation of Societies for Ultrasound in Medicine and Biology (EFSUMB-criteria), based on -among others- the years of experience, the hours of training and the total number of ultrasound scans performed by the operator [20].

The MRI will be performed at 1.5 Tesla (Intera; Philips Medical Systems, Best, The Netherlands). Conventional 2D T2-weighted sequences in three planes (sagittal, coronal, axial) and 3D T1-weighted sequences in the axial plane will be supplemented with diffusion weighted sequences. The MRI scans will be examined by 2 independent radiologists with experience in MRI, and who are blinded to the outcome of the subjective assessment. Furthermore, a blood sample will be taken for translational research purposes from patients with a mass that cannot be classified by simple rules. These serum samples will be stored for future biomarker studies.

A secured online Case Report Form (CRF) and database (‘MACRO’) are created in which the demographic data, ultrasound data and MRI data will be stored.

### Management of the mass

The RMI is currently considered the standard diagnostic procedure. Therefore, in case of an RMI outcome of less than 200 – i.e., when a malignancy is not suspected- the mass is currently managed conservatively or with laparoscopy. In case of an RMI outcome of 200 or more the patient is managed together with a gynecological oncologist from the center hospital for comprehensive surgical staging and cytoreductive (debulking) surgery, according to prevailing guidelines.

However, it can be foreseen that conflicting results can occur between the RMI and the simple rules combined with the second stage test. If this is the case an individual risk assessment will be made and all conflicting results will be discussed with the patient, after which a gynecological oncologist will be consulted when deemed necessary. Although the RMI currently is considered standard care, we believe that conflicting results cannot be ignored.

### Reference standard

Histopathology is the clinical reference standard used in this study. Therefore, histology of the surgically removed adnexal masses is necessary. The resected masses will be classified according to the World Health Organization guidelines for histology [21]. The pathologist will not have access to the results of the index tests. The specimens obtained for histology will be stored for 30 years, according to prevailing guidelines from the Dutch society for pathology [22].

### Ethics and dissemination

The Medical Research Ethics Committee of the Maastricht University Medical Centre in The Netherlands has provided ethical approval for the conduct of the study. Written informed consent will be obtained from all patients before enrollment. The results of the study will be disseminated through international gynecological, radiological and/or oncological peer reviewed publications and conference presentations.

### Analysis

#### Sample size calculation

The RMI has a relatively low sensitivity (75–80 %). By reducing the amount of false negative test results the patient will benefit most in terms of prognosis and survival. For the calculation of the sample size we therefore focused on sensitivity as the most important primary objective.

This study is designed to have an 80 % power to detect an increase of sensitivity from 79 % for the RMI to 91 % for the two-step triage test with a two-sided α value of 0.05. Using a matched pair design, it can be estimated that the total discordance between the preoperative diagnosis and the final diagnosis based on histology, obtained by surgery, is 20 % (16 % and 4 % respectively). Based on McNemar’s test a minimum sample size of 97 women with a malignancy is required [23]. Taking a prevalence of malignancy of 40 % into account, the study will need 243 patients in total. To allow for loss of power of 10 % a total of 270 women will be enrolled in the study.

#### Statistical analysis

We will use McNemar’s test to determine the statistical significance of differences in sensitivity and specificity between the RMI and the two-step test. For statistical purposes borderline tumors will be classified as malignant tumors. The 95 % confidence intervals for sensitivity and specificity will be obtained using Wilson’s interval method [24].

Receiver operating characteristic (ROC) curves will be generated using Medcalc software version 12.7.7.0 (MedCalc Software bvba, Ostend, Belgium) to illustrate the predictive value of the chance of malignancy when using the RMI, simple rules, subjective assessment and MRI. The method described by DeLong et al. will be used for the calculation of the difference between two AUCs [25].

For the subgroup analysis of pre- and postmenopausal women stratification will be applied.

The interobserver-agreement will be evaluated with Cohen’s kappa (kappa values of 0.81–1.0 indicate very good agreement, kappa values of 0.61–0.80 good agreement, kappa values of 0.41–0.60 moderate agreement, kappa values of 0.00-0.40 poor agreement) [26].

Two-tailed *P*-values of ≤ 0.05 will be considered statistically significant for all statistical comparisons.

The economic evaluation will explore the potential cost-effectiveness of the triage test compared to RMI. Incremental cost-effectiveness is expressed as difference in the number of correct diagnosis (i.e., either true positive or true negative for malignancy based on histology) between both methods. With the usual care (RMI) 83 % of all adnexal masses are diagnosed correctly, versus 96 % with simple rules. The consequences of the difference in correct diagnosed patients between the various test are recharged in the treatment (costs of surgical management and hospital stay following surgery) and will therefore lead to a difference in cost-effectiveness.

## Discussion

Currently the RMI is the most frequently used method to distinguish benign from malignant adnexal masses. However, because of its low sensitivity (75–80 %), malignant tumors will be wrongfully diagnosed as benign in a substantial amount of cases. Subjecting these patients to a laparoscopy can induce spill of cyst fluid, which will deteriorate the prognosis of the patient [27].

The aim of this study is to compare the diagnostic accuracy of different diagnostic methods for differentiating benign from malignant adnexal masses. We will test the hypothesis that a two-step triage test consisting of simple rules, if necessary followed by subjective assessment or MRI, will have better diagnostic accuracy than the RMI and therefore will improve the management of women with adnexal masses. Furthermore, a more correct diagnosis will avoid unnecessary costs due to inappropriate or repeat operations. However, unacceptable high costs due to the use of multiple technical examinations should also be avoided. Therefore, we will also examine the cost-effectiveness of changing the diagnostic procedure.

Indirect, we hope to achieve a decrease in peri-operative morbidity and increase of quality of life by diminishing overtreatment, and also an increase in five-year disease-free and overall survival due to improved referral to a gynecological oncologist. However, this study is not powered for these analyses. As such, we will not be able to draw any conclusions with regards to these endpoints.

This study would be the first study that investigates the role of simple rules as a triage test in a geographic referral system. When the hypothesis is confirmed, the results of this study could have major effects on current guidelines and implementation of the triage test in daily clinical practice could be possible. Hospitals will be able to use either simple rules in combination with subjective assessment or in combination with MRI, depending on the expertise present and the sources available in each hospital or region.

## Abbreviations

RMI: Risk of malignancy index
MRI: Magnetic resonance imaging
ROC curves: Receiver operating characteristic curves
